# Integrating Electronic Patient-Reported Outcome Measures into Routine HIV Care and the ANRS CO3 Aquitaine Cohort’s Data Capture and Visualization System (QuAliV): Protocol for a Formative Research Study

**DOI:** 10.2196/resprot.9439

**Published:** 2018-06-07

**Authors:** Diana Barger, Olivier Leleux, Valérie Conte, Vincent Sapparrart, Marie Gapillout, Isabelle Crespel, Marie Erramouspe, Sandrine Delveaux, Francois Dabis, Fabrice Bonnet

**Affiliations:** ^1^ University of Bordeaux, ISPED Inserm, Bordeaux Population Health Research Center, team MORPH3EUS UMR 1219, F-33000 Bordeaux France; ^2^ Centre de Recherche et Développement en Informatique Médicale University of Bordeaux Bordeaux France; ^3^ CHU de Bordeaux COREVIH Nouvelle Aquitaine Bordeaux France; ^4^ AIDES Nouvelle Aquitaine Bordeaux France; ^5^ CHU de Bordeaux Saint-André Hospital Bordeaux France

**Keywords:** patient-reported outcomes, HIV, patient-centered care, health-related quality of life, patient-generated health data

## Abstract

**Background:**

Effective antiretroviral therapy has greatly reduced HIV-related morbidity and mortality, dramatically changing the demographics of the population of people living with HIV. The majority of people living with HIV in France are well cared for insofar as their HIV infection is concerned but remain at risk for age-associated comorbidities. Their long-term, potentially complex, and growing care needs make the routine, longitudinal assessment of health-related quality of life and other patient-reported outcomes of relevance in the current treatment era.

**Objective:**

We aim to describe the development of a Web-based electronic patient-reported outcomes system for people living with HIV linked to the ANRS CO3 Aquitaine cohort’s data capture and visualization system (ARPEGE) and designed to facilitate the electronic collection of patient-reported data and ultimately promote better patient-physician communication and quality of care (both patient satisfaction and health outcomes).

**Methods:**

Participants who meet the eligibility criteria will be invited to engage with the Web-based electronic patient-reported outcomes system and provided with the information necessary to create a personal patient account. They will then be able to access the electronic patient-reported outcomes system and complete a set of standardized validated questionnaires covering health-related quality of life (World Health Organization's Quality of Life Instrument in HIV infection, named WHOQOL-HIV BREF) and other patient-reported outcomes. The information provided via questionnaires will ultimately be presented in a summary format for clinicians, together with the patient’s HIV care history.

**Results:**

The prototype of the Web-based electronic patient-reported outcome system will be finalized and the first 2 formative research phases of the study (prototyping and usability testing) will be conducted from December 2017 to May 2018. We describe the sequential processes planned to ensure that the proposed electronic patient-reported outcome system is ready for formal pilot testing, referred to herein as phases 1a and 1b. We also describe the planned pilot-testing designed to evaluate the acceptability and use of the system from the patient’s perspective (phase 2).

**Conclusions:**

As the underlying information technology solution, ARPEGE, has being developed in-house, should the feasibility study presented here yield promising results, the panel of services provided via the proposed portal could ultimately be expanded and used to experiment with health-promoting interventions in aging people living with HIV in hospital-based care or adapted for use in other patient populations.

**Trial Registration:**

ClinicalTrials.gov NCT03296202; https://clinicaltrials.gov/ct2/show/NCT03296202 (Archived by WebCite at http://www.webcitation.org/6zgOBArps)

**Registered Report Identifier:**

RR1-10.2196/9439

## Introduction

### Background

The advent of effective antiretroviral therapy (ART) in 1996 in resource-rich settings led to a sharp and rapid decline in AIDS-related deaths [1]. In the following years and now decades, improved treatment options have normalized the survival of people living with HIV (PLWH) [2]. For PLWH to benefit fully from ART, they must be engaged in the continuum or cascade of HIV care. In other words, they must be diagnosed early, linked and retained in care, and receive and adhere to effective therapy [3]. The Joint United Nations Programme on HIV and AIDS’ 90-90-90 targets aimed at ending HIV as a public health threat by 2030 are premised on this continuum of care [4]. They call for diagnosing at least 90% of people living with HIV, getting at least 90% of those who are diagnosed on ART, and achieving viral suppression in at least 90% of those who are treated. In settings where the 90-90-90 targets have already been achieved, Lazarus and colleagues have argued that the ultimate goal of HIV care should be to improve health-related quality of life (HRQoL) and have thus proposed a fourth 90%: “achieving good health-related quality of life among 90% of those who are successfully treated for HIV” [5].

This fourth 90% reflects the current needs of the population of PLWH in much of Western Europe, including France, where HIV has become a chronic condition over the most recent decade. The 2013 French HIV Treatment Guidelines first called for addressing the health of PLWH as understood by the World Health Organization, meaning as a “state of complete physical, mental and social well-being and not merely the absence of disease or infirmity” [6]. The concept of HRQoL comes from this definition of health and has become especially relevant to those living with a chronic or recurrent illness.

HRQoL is a common patient-reported outcome (PRO). PROs may be used at the population level for research and to improve health care quality and at the individual patient level to support clinical decision-making and ensure the efficient use of resources. However, despite these potential benefits to both clinicians and patients, PROs have yet to be routinely collected or systematically used in routine care by clinicians [7]. This is due to logistical, methodological, and attitudinal barriers [7]. Some of the first studies on the use of PROs in clinical practice have yielded mixed results. There is strong evidence that having patients complete a self-assessment before a medical visit can facilitate communication about HRQoL [8-13]. Yet, the body of evidence on whether this type of exercise alters patient management, affects outcomes, or improves HRQoL or satisfaction is less well understood [14]. Greenhalgh et al have pointed to the lack of theory-driven approaches used to evaluate the use of PRO measures in routine clinical practice and propose that the mechanisms by which the proposed intervention is hypothesized to affect patient outcomes be clearly delineated [15]. Others like Basch et al have argued that the proliferation of robust survey methods coupled with computerized technologies has provided a potentially viable means of collecting this information and ideally integrating it into electronic medical records (EMR) [16]. Computerized and touch-screen technology can substantially facilitate data collection compared with paper forms, eliminating data-entry and scoring time, and therefore decrease staff burden. Yet, often EMRs and clinical research database systems have been designed to allow for data entry from study staff, making the collection of PROs challenging in some care or research settings [17].

In the context of HIV cohort research, The University of Washington HIV Cohort was among the first to experiment with the routine computerized collection of patient-based measures. Crane et al described efforts to institute the routine collection of electronic PROs (ePROs) in HIV care, concluding that it was both promising for research and clinical care [18]. Whereas Kozak et al highlighted some of the challenges of capturing high-quality data in routine care and the limitations of data recorded in patients’ paper medical or EMR [19]. They note that the demands of clinical care and patients’ willingness to disclose sensitive information may compromise the comprehensiveness and the quality of data captured via EMRs. In their 2012 study, conducted at the University of Alabama at Birmingham 1917 HIV/AIDS clinic, they compared self-reported and EMR data and looked at the association between substance abuse, depression, and poor ART adherence in PLWH. Not only did authors document significant differences in the prevalence of self-reported vs EMR-documented substance use and depression, but they found that the self-reported rather than EMR-documented measures were better correlated with poorer ART adherence [19]. This research suggests that ePRO are an alternative and potentially more reliable means of data capture for sensitive domains such as substance use. Furthermore, ePROs may help clinicians identify problems at the time of care, as demonstrated by Lawrence et al [20]. As part of the same initiative at the 1917 HIV/AIDS Clinic, ePROs were used to detect suicidal ideation and trigger an automated page to predetermined clinic personnel who completed more detailed self-harm assessments [20].

### Objectives

This paper outlines the formative research protocol being undertaken to develop a Web-based system to collect ePROs linked to the existing data capture infrastructure for those in HIV care in southwestern France. The first aim of the ePRO system is to expand and improve the data collection for the ANRS CO3 Aquitaine Cohort of PLWH being followed up in the 13 public hospitals in the region. The second aim is to make this information available to clinicians in a convenient format together with patients’ locally developed, HIV-specific EMR. We describe the sequential process planned to ensure that the proposed ePRO system is ready for formal pilot testing, referred to herein as phases 1a and 1b. We also describe the planned pilot testing designed to evaluate the acceptability and use of the ePRO system from the perspective of patients (phase 2). We have outlined the hypothesized changes induced by the inclusion of these data in a locally developed HIV-specific EMR, which is currently being developed.

## Methods

### Study Designs

The ANRS CO3 Aquitaine cohort is an open, prospective hospital-based cohort. The proposed research was conceived as an ancillary study to the cohort. This protocol reports on the sequential study design from the prototyping (phase 1a) and usability testing (phase 1b) to piloting (phase 2). Phases 1a and 1b rely on mostly qualitative methods. Perspectives of the patient will be assessed and barriers to and facilitators of implementation identified through usability testing. The second phase of the research will initially be based on a cross-sectional study design with the ultimate aim of collecting these data longitudinally (at least once a year) and systematically via the revised ePRO system.

### Platform Design

Clinical and laboratory data from medical records have been collected systematically as part of routine care by a team of clinical research associates/technicians from 13 clinics/hospitals throughout the Aquitaine region since the 1980s and via a locally developed information technology (IT) solution, ARPEGE, since 2013. ARPEGE is a secure Web-based data capture and visualization system developed with Microsoft ASP.NET (WebForm). Data are stored within a Microsoft SQL Server 2014-based data management system. A responsive Web-based platform has been designed for patient follow-up within the existing infrastructure of the ANRS CO3 Aquitaine Cohort. This IT solution was originally developed to meet the data collection requirements of the ANRS CO3 Aquitaine Cohort. Unlike the hospital’s EMR, which did not allow for data to be visualized nor used for research, ARPEGE provides HIV physicians with patients’ medical histories. Its interoperability with the surrounding health information system infrastructure has evolved to allow laboratory data to be downloaded from the Bordeaux University Hospital’s laboratory medicine information system, which includes results of all tests performed as part of hospital-based care. The proposed QuAliV ePRO system expands upon this IT solution by developing a flexible interface for the Web-based collection of ePROs both in a hospital setting and beyond (in the patient’s home) with a special focus on the presentation of individual patient’s results. The inclusion of administrative data from the Program for Medicalizing Information Systems and clinical data from the hospital’s EMR is planned but has not yet been completed.

### Initial Website Specifications

The primary feature of the ePRO system is the survey feature due to the platform being nested within a longstanding hospital-based cohort study. The first feature is to facilitate data collection on HRQoL and its main determinants via validated electronic questionnaires. The content of the patient interface is based on current treatment guidelines for people being treated for HIV and associated comorbidities [6]. French guidelines recommend an annual checkup, during which a number of issues should be addressed by the HIV physician according to the patients’ age and sex. According to the taxonomy of applications of PROs in clinical practice laid out by Greenhalgh, the proposed system aims to optimize this checkup by having the patient complete a standardized self-reported questionnaire before the visit [10]. The proposed ePRO system relies on a selection of validated questionnaires that were mostly already available in French. The questionnaires have already been evaluated individually according to their psychometric properties, administration method, and length. The following areas are covered by the ePRO system, broken up into thematic modules covering: socioeconomic status and individual social and material deprivation (Evaluation de la Précarité et des Inégalités de santé dans les Centres d’Examens de Santé [EPICES]) [21], multidimensional quality of life (WHOQOL-HIV BREF) [22], treatment burden (Treatment Burden Questionnaire) [23], physical activity (The Short Version of the International Physical Activity Questionnaire [IPAQ]), alcohol use and screening for at-risk drinking behavior (Alcohol Use Disorders Identification Test Consumption [AUDIT-C], Fast Alcohol Consumption Evaluation [FACE]) [24], tobacco and nicotine use and screening for tobacco dependency (Fagerström), cannabis (Cannabis Abuse Screening Test [CAST]) and drug use, and finally, depression (Patient Health Questionniare [PHQ-9]) [25]. The system also allows patients to report any other treatment-related issues in a free-text field. Where applicable, we have followed the recommendations put forth by the International Society for Pharmacoeconomics and Outcomes Research ePRO Task Force on adapting paper-based instruments to ensure that data produced are equivalent or superior to those generated from paper-based administration methods [26]. It should be noted that the choice of questionnaires for the initial prototype is intentionally more exhaustive than the anticipated final version, as we do not know whether the questionnaires selected will be adequate in terms of their psychometric properties. This will be verified during the pilot phase.

We have planned additional IT security measures including the encryption of email addresses using the Advanced Encryption Standard encryption algorithm with a key length of 256 bits. Advanced Encryption Standard encryption technology is currently one of the most secure. Passwords will be encrypted by the BCrypt algorithm, which is recognized as being at the cutting edge of hash chain technology. Furthermore, passwords created by the user must contain at least 8 alphanumeric characters including at least 2 special characters, a capital letter, and a number that must be changed every year. The unique study-specific identification number will contain 8 randomly defined alphanumeric characters.

### Preimplementation (Phases 1A and 1B)

The IT solution, ARPEGE, has been made available in hospital-based HIV care centers since 2013. Its use is facilitated by research assistant technicians. To inform the implementation strategy, taking into account the facilitators and barriers faced by users, a preimplementation assessment identifying those factors crucial to implementation success or failure will be conducted before determining the final implementation procedure.

### Phase 1A: Prototyping (Eliciting Feedback on Initial Specifications)

On the basis of the above specifications, a preliminary version of the interface will be constructed and presented to patients to elicit their feedback. The aim of the preliminary qualitative interviews is not to rigorously evaluate the website’s performance but to obtain information that could be used to develop the interface and prepare it for formal pilot testing. Using a semistructured interview guide, we will interview a convenience sample of 10 HIV patients of varying ages, transmission groups, and genders. During the interview, the interviewer will present a mock-up of the Web-based patient interface and describe its proposed functions to each participant.

### Phase 1B: Usability Testing

Usability measures to what extent a person can use a system for its goal effectively, efficiently, and satisfactorily [27]. Usability testing will be conducted on the prototype of the ePRO system according to guidelines from the website Usability.gov [28]. A convenience sample of 10 patients will be recruited and interviewed in an outpatient hospital setting. This sample is considered adequate to evaluate whether the website is ready for planned, more rigorous, pilot testing [29]. Eligible patients, identified by clinical staff, will be approached for the study before their scheduled visit. During usability testing, patients will access the website and test its features, including the site login, survey completion, and review of results. Patients will “think aloud” as they complete the login process and survey and complete a semistructured interview about the ease of use and completion, presence of mistakes or problems, user satisfaction, likes/dislikes, and their willingness to use it regularly before visits. Efficiency (eg, time it takes to complete tasks) will also be monitored. Finally, participants will also be asked to complete the System Usability Scale (SUS), a validated 10-item scale with Likert-scaled responses ranging from “strongly agree” to “strongly disagree” and a summary score [28].

The findings from this first phase (1a and 1b) will inform the second phase of the study, which will extend the implementation of the proposed patient interface in a limited number of hospitals and aims to evaluate its acceptability and use.

### Phase 2: Pilot Testing

#### Proposed Setting

The study will be carried out in the ANRS CO3 Aquitaine Cohort, an open, prospective, hospital-based cohort of PLWH followed-up in 13 clinics in southwestern France. The pilot testing will take place in 3 of them, selected to reflect variations in resources (human and material) or geographic setting (rural vs urban clinics). We aim to assess the acceptability of the proposed system in different clinical contexts to eventually offer center-specific adjustments to the proposed implementation procedures.

#### Inclusion and Exclusion Criteria (Phase 2)

As this study will be nested within a longstanding existing hospital-based cohort of PLWH, those invited to participate in this study must meet the cohort’s eligibility criteria: aged 18 years or older, confirmed HIV-1 diagnosis, and having signed a consent form. Access to a personal email account and the internet via either a computer or smartphone in a private setting will be verified by the clinician before the participant is invited to engage with ePRO system. Patients who express an interest in completing a self-reported questionnaire but lack either a personal computer or smartphone and/or reliable internet access will be provided with a paper version questionnaire or, depending on the study center, invited to use a study-specific electronic tablet (Samsung Galaxy Tab S2).

#### Patient Selection and Recruitment

**Figure 1:**
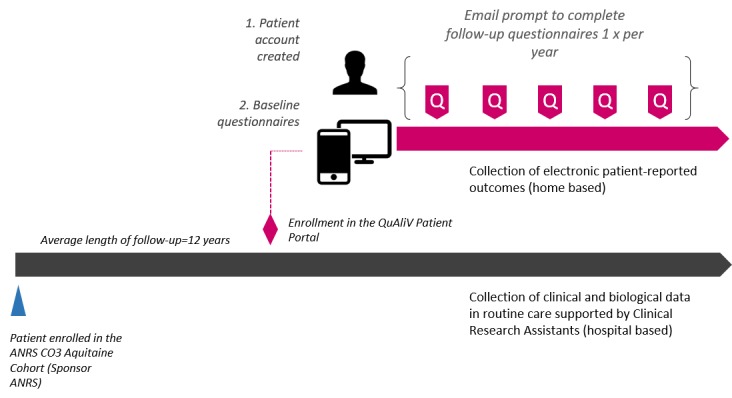
Integration of the QuAliV patient portal in the ANRS CO3 Aquitaine Cohort.

Patient selection and recruitment will be done in tandem with planned administrative changes to the cohort and will take place during routine care. The standard operating procedures detailing the new procedures for including participants in this component of the cohort have been developed during successive team meetings. Before each visit, on-site research assistants will provide clinicians with a study-specific randomly generated patient identifier. Clinicians will invite patients to participate in the ANRS CO3 Aquitaine cohort’s new research initiative at the time of the consultation. Figure 1 outlines the integration of the QuAliV patient portal in the ANRS CO3 Aquitaine Cohort.

Once the eligibility criteria have been verified, if the patient wishes to participate in the study, he/she will be provided with a patient-oriented brochure developed specifically for those interested in engaging with the system. The study-specific identifier required for participants to create their accounts will then be noted on a detachable part of the patient-oriented brochure for easy reference. This study-specific identifier is required to create an account via ARPEGE. It allows for the patient account and the self-reported data to be linked to the existing clinical data capture and visualization system (ARPEGE).

As the patients could be accessing the website from their phones or from their home computers, the section of the brochure for noting the study-specific identifier will be detachable, allowing the study participant to leave the brochure at the hospital for the sake of confidentiality (Multimedia Appendix 1). To monitor study enrollment and ascertain whether the proposed system is acceptable to users, enrollment will be tracked by the centers.

Eligible participants will be directed to the study website where they will be provided with additional details about the research initiative and its aims. The website will provide additional information to “recruited” patients, encouraging them to take a more active role in his or her HIV care and well-being. The patient will be redirected to the account creation page, powered by ARPEGE. To ensure that the participant created his/her account successfully, he/she will be asked to enter his/her email twice together with the unique patient identifier. The patient will then be asked to confirm his/her email address before he/she can access the patient portal. Metadata will be monitored to identify any bottlenecks during the pilot phase.

#### Study Population

The cohort’s “active follow-up” is defined as patients who have been seen over the course of the previous year either at a hospital-based consultation or been hospitalized. In 2016, approximately 4480 patients were actively being followed-up in the cohort. The average length of follow up is 12 years post HIV diagnosis. In total, 27.95% (1252/4480) of the cohort is female and mean age is 51 years (SD 11 years). The majority of the cohort contracted HIV through sex (41.93% (1881/4486) are men who have sex with men and 37.09% (1664/4486) are heterosexuals) and 12.75% (572/4486) through injection drug use. Moreover, 20.60% (923/4480) of those in active follow-up have been diagnosed with AIDS, 26.70% (1010/3745) are overweight, and 8.62% (323/3745) are obese. In addition, 43.71% (1831/4189) report being current smokers.

#### Statistical Analysis

Feedback on initial specification from patients will be evaluated qualitatively during phase 1a. During phase 1b, in addition to qualitative feedback provided using the “think aloud” approach and semistructured interview, we will define success in usability a priori as SUS score reaching a ceiling effect: with a minimum score of 70 as the generally accepted cutoff usability rating for “good” [27]. For each measure, we will also calculate the percentage of completed items by the total number of items for each PROs module.

We will monitor eligibility, QuAliV numbers issued, accounts created, and initial questionnaires completed within 1 month of the visit. The following process indicators will be used to assess acceptability:

- The proportion of people who refused to participate in the study
- the proportion of those who received information but failed to create an account
- The proportion of those who created an account but failed to complete the questionnaires

To assess use, the main outcomes of interest of the phase 2 study are the overall participation rate (proportion of those who created an account and completed the assessment) implemented as a pilot and the participation rates based on readily available personal, demographic, and treatment-related factors. Differences based on age, sociodemographic characteristics (including rural vs urban), and clinic and transmission groups will be evaluated using the chi-square test. Determinants of use will be evaluated using logistic regression methods.

As all the questionnaires will be used in an electronic form, the psychometric properties of the instruments included in the patient portal will also be verified. We will be especially attentive to the presence of floor and ceiling effects (60% of responses in extreme categories). We will also monitor the time it takes to complete the questionnaire as a further indicator of feasibility. The dimension of HRQoL measured by the instruments will be assessed using confirmatory factor analysis.

Finally, we will use the pilot phase to verify the distribution of the main outcome of interest (HRQoL) of our epidemiological study, seeking to measure both the prevalence and the determinants of poorer HRQoL in PLWH in the current HIV treatment era. We will use this initial sample to calculate the required sample size and plan for the scale up of the platform in all of the participating hospitals/clinics in the region.

#### Ethical and Legal Aspects

The implementation of this study called for an amended version of the cohort protocol to be submitted to an ethics committee. This amendment entailed a detailed description of the content of the questionnaires included in the ePRO system, the content of patient-oriented brochure and the external patient-oriented website. Approval was granted in August 2017.

As the implementation of this system requires patients to use their email addresses to create their personal accounts, an amendment to the regulatory authorizations previously granted to the cohort by The French National Commission on Informatics and Liberty, an independent administrative regulatory body charged with ensuring that data privacy laws are applied to the collection, storage, and use of personal data, was requested in late 2017 and approval was granted on March 12, 2018.

## Results

Seed funding was granted by France REcherche Nord&Sud Sida-hiv Hépatites (ANRS) in 2017 via the CSS-5 call in January, 2017 and additional staff recruited in June 2017 to develop the ePRO system’s infrastructure. DB was awarded a 36-month “young researcher” grant from Sidaction to design and conduct this study within the ANRS CO3 Aquitaine cohort as part of her doctoral research.

The development of the prototype of QuAliV ePRO system and the first 2 phases of the study will be conducted between December 2017 and May 2018. The results from phase 1 will ultimately inform the implementation of the pilot project. Efforts to integrate data generated from the ePROs system into a HIV-specific EMR will begin in April 2018 as part of the next phase of APREGE’s development. Enrollment of participants is planned in June 2018.

## Discussion

Although France boasts a robust public health and epidemiological surveillance system, its cohorts relied, until recently, on paper-based data collection methods. The Aquitaine cohort, launched in 1987, transitioned to an electronic Case Report Form supported by center-based clinical research technicians in 2013. The relatively recent transition to an electronic data capture and visualization system has made the collection of ePROs in hospital-based cohort studies of PLWH conceivable and timely in light of the current HIV care paradigm in France. The introduction of the proposed ePRO system and updated physician HIV-specific EMR, presenting a summary of patients’ clinical, laboratory, and self-reported records, will imply changing both patient behavior and daily clinical practice.

**Figure 2:**
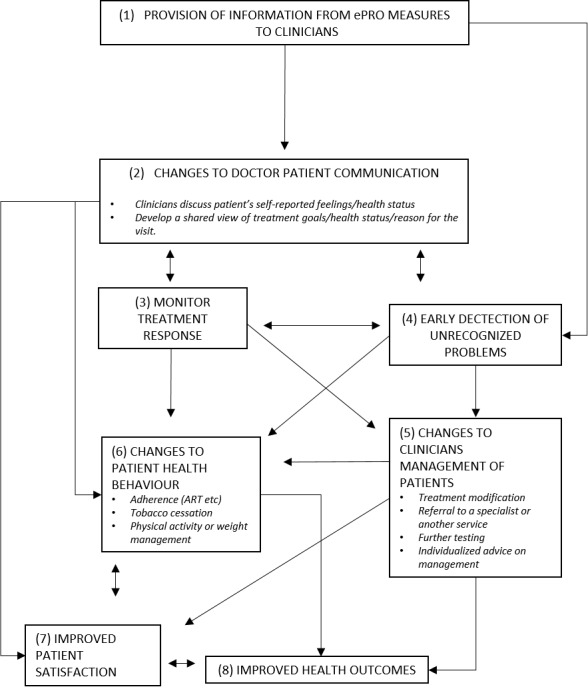
Hypothesized change to clinical decision making resulting from use of the ePRO system, adapted from Greenhalgh et al.

In line with recommendations put forth by Greenhalgh and colleagues, we have diagrammed the hypothesized mechanisms by which this patient ePRO system is designed to promote improved patient-physician communication (Figure 2, adapted from Greenhalgh et al) [15]. The results of the self-reported questionnaires will be summarized for clinicians in a convenient format developed in collaboration with end users. We hypothesize that providing this information can improve communication and, thus, lead to better quality of care (both patient satisfaction and health outcomes). Presenting this information will also allow HIV physicians to monitor the patient’s response to treatment over time (ART and treatment for associated comorbidities) and/or detect issues that may have previously gone unnoticed (eg, a change in employment status, living conditions, addictions, a lack of social support, depression, and/or a decline in HRQoL). We hypothesize that physicians will also be better equipped to discuss health-promoting behaviors such as exercise or smoking cessation, adjust treatment regimens, or refer patients to a specialist or allied health professionals (eg, therapist, dietician, social worker).

As the underlying IT solution, ARPEGE, was developed in house, should the phases 1a and 1b and phase 2 studies, presented here, yield promising results, the panel of services provided via the proposed ePRO system could ultimately be expanded. For example, continuous patient education/coaching for better self-management, similar to interventions that have been implemented for other chronic conditions (diabetes, heart disease, etc), could be offered, as could decentralized models of care and/or facilitated communication with one’s general practitioner. The adoption of these different services could ultimately be the aim of future experimental research in this patient population aging with HIV. Alternatively, the proposed system, designed for outpatient hospital-based HIV care, could be adapted for use in other chronic diseases and/or other care settings.

## Appendix

Multimedia Appendix 1 Patient-oriented brochure with detachable coupon.

Multimedia Appendix 2 Conclusions of External Peer-review (English Translation).

## Abbreviations

ART: antiretroviral therapy
EMR: electronic medical records
ePRO: electronic patient reported outcome
HRQoL: health-related quality of life
PLWH: people living with HIV
PRO: patient-reported outcome
WHO: World Health Organization
