# Supplementary figures and images for: Integrating Electronic Patient-Reported Outcome Measures into Routine HIV Care and the ANRS CO3 Aquitaine Cohort’s Data Capture and Visualization System (QuAliV): Protocol for a Formative Research Study
Source: JMIR Res Protoc. 2018 Jun 7;7(6):e147. doi: 10.2196/resprot.9439 (PMC6013715; doi:10.2196/resprot.9439)

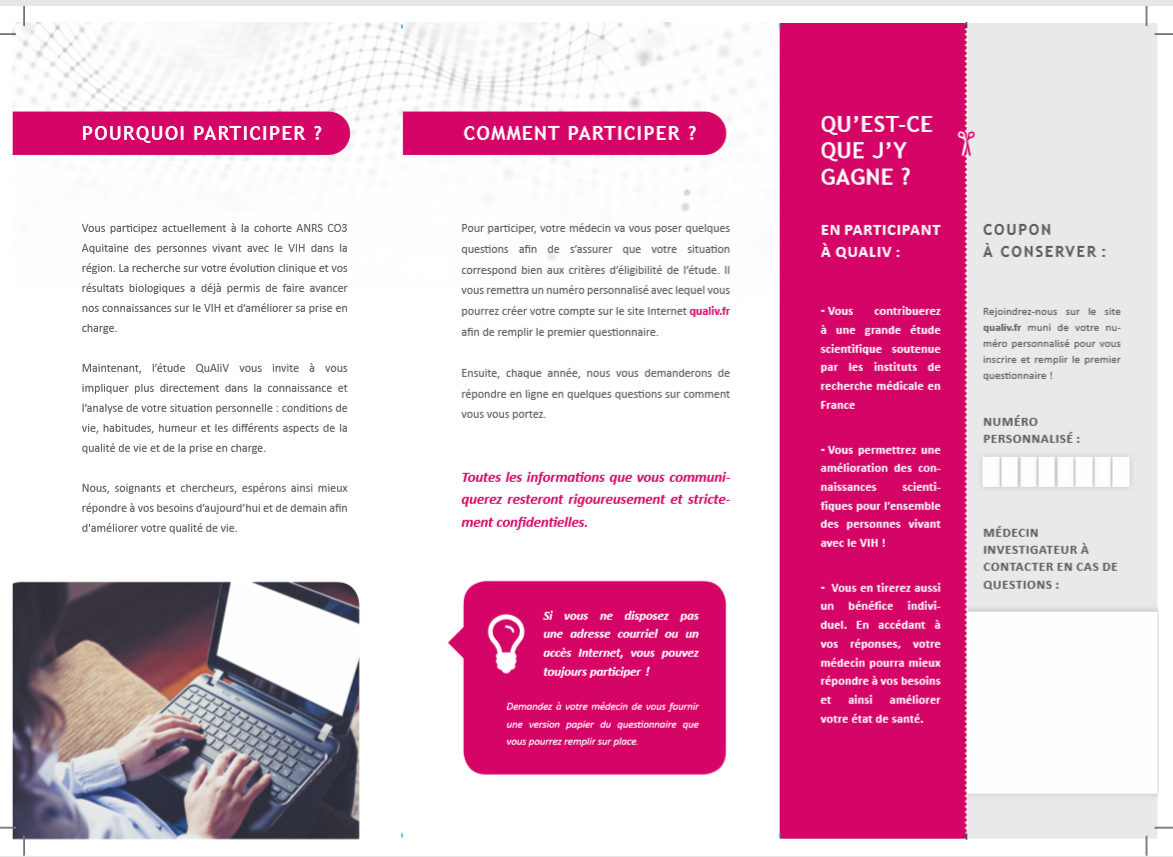

Supplement: Multimedia Appendix 1 [file resprot_v7i6e147_app1.png]
