# Supplementary material for: Integrating Electronic Patient-Reported Outcome Measures into Routine HIV Care and the ANRS CO3 Aquitaine Cohort’s Data Capture and Visualization System (QuAliV): Protocol for a Formative Research Study
Source: JMIR Res Protoc. 2018 Jun 7;7(6):e147. doi: 10.2196/resprot.9439 (PMC6013715; doi:10.2196/resprot.9439)
Supplement: Multimedia Appendix 2 [file resprot_v7i6e147_app2.pdf]

Conclusion CSS

CSS 5 - Research in Public Health and Humanities and Society

Project leader: BONNET Fabrice

Project title: **Feasibility, Acceptability, and Implementation of an electronic patient-reported outcomes (ePRO) system for people living with HIV (PLWH) in routine care in the Aquitaine region of France**

This application for seed funding (type of grant) resembles a small research project grant application. It is nevertheless very relevant, with ambitious and much needed data collection efforts. Qualitative methods should be further explained as well as the number of people who will participate in the surveys.

Highly ranked by the CSS 5, the project has been awarded funding.
